# Obstetric Brachial Plexus Palsy: The Mallet Grading System for Shoulder Function—Revisited

**DOI:** 10.1155/2014/398121

**Published:** 2014-01-05

**Authors:** M. M. Al-Qattan, A. A. F. El-Sayed

**Affiliations:** Department of Surgery and Obstetrics, King Saud University, P.O. Box 18097, Riyadh 11415, Saudi Arabia

## Abstract

The Mallet grading system is a commonly used functional scoring system to assess shoulder abduction/external rotation deficits in children with obstetric brachial plexus palsy. One feature of the Mallet score is that each grade is translated into certain degrees of deficiencies in both shoulder abduction and external rotation. The aim of the current study is to investigate the percentage of children in which the Mallet score could not be applied because of a discrepancy between the deficiency of shoulder abduction and shoulder external rotation. The study group included 50 consecutive unoperated older children (over 5 years of age) with Erb's palsy and deficits in shoulder movements. The Mallet score could be applied in 40 cases (80%). In the remaining 10 cases (20%), the Mallet score could not be applied either because shoulder abduction had a better grade than the grade of shoulder external rotation (*n* = 7) or vice versa (*n* = 3). It was concluded that documenting the deficits in shoulder abduction and external rotation are best done separately and this can be accomplished by using other grading systems.

## 1. Introduction

The most common secondary deformity in obstetric brachial plexus Erb's palsy is seen at the shoulder; the most common shoulder deformity is the deficiency of shoulder abduction/external rotation [1].

There are different methods of scoring shoulder abduction/external rotation of the shoulder such as the Toronto muscle grading system [2], the King Saud University grading system [3], and the Mallet grading system (Table 1). The Mallet grading remains the most commonly used system in several obstetric brachial plexus centers [4–6]. One feature of the Mallet score is that each grade is translated into certain degrees of deficiencies in both shoulder abduction and external rotation. The authors noted that the Mallet score could not be applied to several children with obstetric ERb's palsy mainly because there is a discrepancy in the Mallet grade of shoulder abduction and the grade of shoulder external rotation.

The aim of this study is to investigate the percentage of children in which the Mallet score could not be applied to assess shoulder function in a series of unoperated older children (over 5 years of age) with obstetric brachial plexus Erb's palsy. Older children were selected because the Mallet score requires special tasks that are difficult to apply to infants and younger children.

## 2. Patients and Methods

Fifty consecutive unoperated children (over 5 years of age) with shoulder deficits were selected for the study. All children were seen at our obstetric brachial plexus clinic [7]. Selection criteria were Erb's obstetric palsy, no previous surgery, age over 5 years of age, active elbow flexion (over 100 degrees) against resistance, and adequate data documenting the shoulder deficits. The Mallet score (Table 1) was documented to assess shoulder function if possible. If the Mallet score could not be applied, the reason for discrepancy between shoulder abduction and external rotation is documented. The percentage of the latter group of children is also calculated.

## 3. Results

The mean age of the study group (*n* = 50) was 8 years (range 5–15 years). All children had cephalic presentation and a history of difficult delivery. Forty-four of these children presented late to our clinic for correction of secondary shoulder deformity. The remaining six children were initially seen in our clinic soon after birth, but no primary exploration was done because children recovered active elbow flexion against gravity prior to 4 months of age. These six cases were then lost for followup for 5–8 years and then presented back to our clinic with the shoulder deficits.

Table 2 summarizes the results. The Mallet score could be applied to assess the shoulder deficits in 40 cases (80%). In these cases, the deficit of shoulder abduction coincided with the deficit of shoulder external rotation in each case according to the criteria put by the score in Table 1. In 10 cases (20%), the Mallet grading system could not be applied either because shoulder abduction had a better grade than the grade of shoulder external rotation (*n* = 7) or vice versa (*n* = 3).

## 4. Discussion

The current study is the first study that assesses that applicability of the Mallet score in children with Erb's palsy. Clinically, the surgeon tailors the indications of surgery depending on the degree of the deficit of every joint motion. However, the Mallet score is frequently used in research papers to assess the combined deficits at both shoulder abduction and external rotation. It is of concern that 20% of children will have a discrepancy in the Mallet score of shoulder abduction and external rotation. Hence, for research papers, it seems that it is more logical to document the deficits in shoulder abduction and the deficits in shoulder external rotation separately; this can be accomplished by using other grading systems [2, 3].

One observation of our results is that none of the 50 children had Grade I shoulder deficit (flail shoulder). A flail shoulder is usually seen in obstetric palsy following breech delivery with C5/C6 avulsion [8]. All our children in our study group had a cephalic presentation.

In seven of the 10 children in whom the Mallet score could not be applied to, shoulder abduction grade was better than the grade of shoulder external rotation. This suggests that the suprascapular nerve sustained a higher grade of injury than the posterior division of the upper trunk. The most unusual case was Case ≠ 7 (Table 2) in whom shoulder abduction was normal (Grade V) while the Mallet grade of shoulder external rotation was only Grade II.

The deltoid muscle is the primary abductor of the shoulder; the infraspinatus muscle is the primary shoulder external rotator. Anatomically, both of these muscles are supplied by the fifth cervical root (C5). However, the nerve fibers supplying the deltoid muscle are carried through the posterior division of the upper trunk, while the nerve fibers supplying the infraspinatus are carried through the suprascapular branch of the upper trunk. Obstetric paralysis is mainly caused by traction of the nerve roots during delivery [9], and severity of intrapartum forces on the roots of the brachial plexus will vary greatly depending on fetal factors (such as fetal macrosomia and fetal presentation), maternal factors (such as the degree of cephalopelvic disproportion), and delivery factors (such as the use of instrumental delivery and maneuvers used to manage shoulder dystocia) [10]. Furthermore, the cadaveric studies of G. A. Brunelli and G. R. Brunelli [11] showed that the direction of force as well as the anatomical position of each root or branch of the brachial plexus will also determine the degree of axonal damage from an externally exerted force on the neck. Since the deltoid and infraspinatus muscles are supplied by two different branches of the upper trunk, the force (and hence the degree of axonal damage) may be different on each branch, explaining the differences in the deficits of shoulder abduction/external rotation in the same patient.

In conclusion, the Mallet score could not be applied to 20% of unoperated children with obstetric brachial plexus palsy because of the discrepancy of the grade of shoulder abduction and the grade of shoulder external rotation in the same patient. This suggests that the intrapartum forces on the posterior division of the upper trunk may vary from the forces on the suprascapular branch of the upper trunk in the same patient.

## Figures and Tables

**Table 1:** The Mallet grading system for shoulder function.

Grade	Description
I	Flail shoulder

II	Active abduction ≤30° Zero degrees of external rotation Hand to back of neck impossible Hand to back impossible Hand to mouth with marked trumpet sign

III	Active abduction 30–90° External rotation up to 20° Hand to back of neck with difficulty Hand to back with difficulty Hand to mouth possible with partial trumpet sign (over 40° of shoulder abduction)

IV	Active abduction over 90° External rotation over 20° Hand to back of neck easy Hand to back easy Hand to mouth easy with less than 40° of shoulder abduction

V	Normal shoulder

**Table 2:** The Mallet grading system applied to 50 children with Erb's obstetric palsy with shoulder deficits.

Group I (*n* = 40): Mallet grading system could be applied to assess the shoulder deficits	Group II (*n* = 10) Mallet grading system could not be applied to assess the shoulder deficits because of discrepancy in the grade of		
		Shoulder abduction grade	Shoulder external rotation grade
Grade I = no cases	Case 1	III	II
Grade II = 13 children	Case 2	III	II
Grade III = 15 children	Case 3	IV	III
Grade IV = 12 children	Case 4	IV	II
Grade V = no cases	Case 5	IV	III
	Case 6	IV	III
	Case 7	V	II
	Case 8	III	IV
	Case 9	II	III
	Case 10	II	III
